# “It’s a proactive intervention instead of a reactive one”: measuring facilitators and barriers regarding readiness to implement a treatment program for infants with neonatal opioid withdrawal syndrome

**DOI:** 10.1186/s12913-023-09734-8

**Published:** 2023-07-14

**Authors:** Leah Holcomb, Caitlin Koob, Rachel Mayo, Elizabeth Charron, Lori Dickes, Windsor Sherrill, Jennifer Hudson

**Affiliations:** 1 Department of Public Health Sciences, 503 Edwards Hall, Clemson University, Clemson, SC 29634 USA; 2 Department of Health Promotion Sciences, Hudson College of Public Health, University of Oklahoma Health Sciences Center, Schusterman Center, 4444 E 41St St, Tulsa, OK 74135 USA; 3 Department of Political Science, 2023 Barre Hall, Clemson University, Clemson, SC 29634 USA; 4 Newborn Services, Prisma Health Upstate, 701 Grove Road, Greenville, SC 29605 USA

**Keywords:** Infants, Opioid use disorder (OUD), Neonatal opioid withdrawal syndrome (NOWS), Healthcare delivery

## Abstract

**Background:**

Managing Abstinence in Newborns (MAiN) is an evidence-based, cost-saving approach to caring for infants at risk of developing neonatal opioid withdrawal syndrome (NOWS). MAiN provides medication management in combination with education and is being implemented in hospitals across South Carolina (SC). This expansion of MAiN throughout the state includes educational training for providers on managing NOWS symptomology and evaluation support for data collection and analysis. This evaluation assessed the readiness of hospitals to implement MAiN by identifying potential barriers and facilitators to early program adoption.

**Methods:**

We used the Consolidated Framework for Implementation Framework (CFIR) to guide the evaluation. As part of the ongoing evaluation of MAiN implementation, brief, structured interviews were conducted with healthcare providers (*n* = 82) at seven hospitals between 2019 and 2022 to learn more about perceived barriers and facilitators to implementation readiness. Two coders independently reviewed all transcripts and used deductive thematic analysis to code qualitative data using Atlas.ti Web using the established CFIR codebook.

**Results:**

We identified barriers and facilitators to implementing MAiN in all five CFIR domains. Providers identified MAiN as an evidence-based, patient-centered model with the flexibility to adapt to patients’ complex needs. Specific champions, external support, alignment with providers’ personal motivation, and an adaptable implementation climate were identified as facilitators for implementation readiness. Barriers included a lack of consistent communication among hospital providers, minimal community resources to support patients and families after discharge, and a lack of provider buy-in early in implementation.

**Conclusions:**

Key barriers and facilitators of MAiN implementation readiness were identified at seven participating hospitals throughout SC. Communication, staff and hospital culture and climate, and internal and external resource were all reported as essential to implementation. These findings could inform the MAiN program expansion in hospitals across SC.

**Supplementary Information:**

The online version contains supplementary material available at 10.1186/s12913-023-09734-8.

## Background

There have been rapid increases in the number of pregnancies affected by opioid use disorder (OUD) and neonatal opioid withdrawal syndrome (NOWS) in the U.S. Nationally, it is estimated that 2.6 to 16.2 of every 1000 infants delivered are affected by NOWS [1]. In South Carolina (SC), rates of NOWS have nearly tripled from three per 1,000 births in 2010 to 28 per 1,000 births in 2018 [2]. There is no nationwide consensus on how to manage NOWS, with the management of affected infants varying between healthcare institutions.

A care delivery program for infants with NOWS—the Managing Abstinence in Newborns (MAiN) program—was established in 2003 by Prisma Health, a tertiary care academic medical center in Greenville, SC, to address the gaps in evidence-based care for this population. Since that time, MAiN has emerged as a novel, evidence-based, cost-saving approach to caring for infants at risk of NOWS [3, 4]. The program includes early initiation of pharmacological therapy for infants at high risk of developing severe NOWS withdrawal symptomology, treatment of NOWS in the nursery rather than higher acuity care settings such as the Neonatal Intensive Care Unit (NICU), comprehensive education for clinical staff regarding NOWS treatment; rooming-in for mothers following delivery to increase early involvement in the infant’s care; and ongoing support from a clinical and evaluation team engaged with the MAiN program [5]. The model also emphasizes the use of medically correct terminology when referring to fetal and neonatal substance exposure, and the importance of ensuring providers do not refer to infants as “being born addicted” but rather opioid-exposed.

**Fig. 1:**
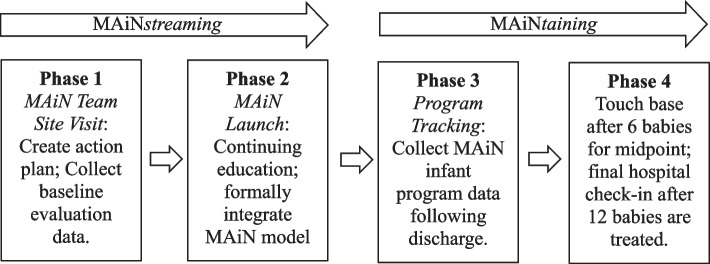
MAiN Implementation Overview. The four phases of MAiN 2.0 include the initial site visit, the formal launch after all continuing education has been completed, ongoing use of the MAiN model until the midpoint evaluation (hospital has treated 6 infants using MAiN) and the final evaluation (12 infants have been treated with MAiN). The baseline evaluation described here occurred during Phase 1

Because of this comprehensive and cost-effective approach to managing NOWS, the SC Department of Health and Human Services provided support to implement MAiN in 10 hospitals throughout SC to treat 120 infant beneficiaries statewide. The MAiN expansion project is ongoing and, to date, has recruited seven hospitals, which are at varying stages of implementation and program maintenance. The baseline interviews described later all occurred at the start of implementation for each of those seven hospitals. Figure 1 provides a visual overview of the MAiN 2.0 implementation process. At the time of baseline interviews described here, all seven hospitals were in Phase 1.

Given that MAiN was developed at a regional medical center with significant resources, ensuring MAiN can be adapted to fit the needs of facilities with limited capacity is vital to implementation success. As part of ongoing efforts focused on MAiN implementation and eventual integration into clinical practices, we used the Consolidated Framework for Implementation Research (CFIR) [6] to identify potential barriers and facilitators to implementing MAiN within SC hospitals. This evaluation is rooted in dissemination of implementation and evaluation findings to a broader audience for improving care delivery to high risk populations [7]. Clinical environments contain many factors that influence the successful implementation of a program. The use of the CFIR model to fully identify barriers and facilitators to the MAiN implementation was determined at the project’s inception. Although CFIR has traditionally been used as an implementation framework, dissemination of MAiN will also utilize the CFIR model. Using CFIR constructs improves finding generalizability and project replication [8]. Here we report the results of those efforts.

## Methods

### Facility selection

Hospitals were recruited through word-of-mouth at monthly SC Birth Outcomes Initiative (BOI) meetings, targeted announcements, and direct contact with the pediatrician who developed MAiN. Interested hospitals were asked to submit an application that included current resources and the average number of infants recently treated for NOWS. Facilities had to meet a clinical threshold of need, defined as a minimum of 12 infants with NOWS treated at that site in the preceding two years, allowing for pre- and post-outcome comparison as well as demonstrating the volume of NOWS care warranted implementation. This threshold was determined from preliminary data each hospital submitted as part of their application. Larger regional hospitals treated an average of 25 infants diagnosed with NOWS in the two years before their application, while smaller rural hospitals treated an average of 6–8 infants with NOWS. The threshold of 12 NAS cases was a reasonable volume that all enrolling hospitals would be able to provide care for in a two-year period.

### Sampling and participants

We recruited 82 healthcare providers at seven participating hospitals. A purposive sampling technique was used wherein the primary champion at each facility invited a unique sample of providers to participate. Baseline interviews were conducted following a brief training session conducted on site at each facility. Training included a comprehensive overview of the MAiN model with a clinician, as well as discussion around NOWS care and major problems anticipated by each hospital’s clinicians. Providers later completed a standardized educational course online which qualifies for six hours of continuing education, including two hours of opioid-specific continuing education [9]. While there was no set sample size, at least eight providers were included from each site to ensure a relatively representative sample. All efforts were made to recruit other clinical and administrative staff to collect distinct viewpoints that described implementation barriers and facilitators at all levels within the hospital. Because this evaluation was not deemed as research, and as such exempt, under the institutional review board standards, written consent was not obtained.

### Interview guide development

The CFIR framework and established codebook informed the development of the interview guide. Using CFIR’s well-defined constructs allowed for the development of brief, flexible questions to meet the evaluation needs for data collection in busy hospital settings [6]. Questions were designed to elicit information on potential barriers and facilitators to implementation and generate information about each hospital's learning environments and organizational culture, and as such drew heavily from the process, inner setting, and innovation characteristic domains. This evaluation was conducted at baseline implementation in each hospital. The interview guide was piloted with two pediatric nursing specialists and then refined and finalized by the study team. A copy of the final questions can be found in Additional file 1.

### Data collection and analysis procedures

Five trained evaluators associated with the project conducted in-person structured interviews with providers that lasted an average of 15 to 20 min and transcriptions were uploaded to Atlas.ti Web [10]. Interviews were conducted on site between 2019 and 2022, occurring at the start of implementation following a training session. Transcripts were typed by each interviewer rather than obtain audio recording to minimize loss of confidentiality. Two research team members independently reviewed transcriptions and applied deductive codes that were a priori identified from CFIR constructs used to develop the interview guide. Data were compared as a single group to ensure provider-type did not compromise confidentiality of responses. Constructs were only included if observed consistently across all seven hospitals. A total of 16 CFIR constructs were identified—four were included from Innovation Characteristics, two from Outer Setting, five from Inner Setting, three from Characteristics of Individuals, and two from Process.

## Results

Participants included 48 pediatric and labor and delivery nurses, 16 physicians, eight pharmacists, six social workers, and four administrators. Results are summarized below according to the five CFIR domains and associated constructs. Table 1 provides a list of identified constructs, a brief description, and identified primary barriers and facilitators.

**Table 1:** Summary of findings by the Consolidated Framework for Implementation Research (CFIR) domains and constructs

**Innovation Characteristics**		
*CFIR Constructs*	*Facilitators*	*Barriers*
*Evidence Strength:* Whether the innovation or program is evidence-based and supported by previous research	MAiN as a standard of care Structured, evidence-based tool Consistent guidance for staff proactive intervention	Staff are not included in the intervention implementation and planning phases
*Relative Advantage:* Whether an innovation is better than other available practices	Uniform approach Localized, consistent care Decreases LOS Reduces staff burden Safer, patient-centered approach Less variability in subjective screening than previous tool	None identified
*Adaptability:* How well a program can be tailored to fit the needs of an individual site	Established tools to guide implementation Standardized protocols Flexibility Measure readiness prior to implementation Identified champions No current standard	Locum physicians Patients’ varying pharmacy access Hesitancy among staff and patients Staff capacity for change Varying hospital size Technical difficulties (i.e. order sets)
*Complexity:* The anticipated difficulty of implementation	Interdisciplinary program Employs patient-centered care	High-risk population
**Outer Setting**		
*CFIR Constructs*	*Facilitators*	*Barriers*
*Patient Needs and Resources of those Served by the Organization:* Needs and resources focused on primary recipients of a new program are essential in examining how well that new program will fit within the current environment	Improved care environment Promotes mother-baby bonding Trained staff Early identification and treatment Staff willingness to adopt new methods	Growing population in need of specialized care Varying parental involvement Lack of appropriate access Unmet social determinants of health needs Limited community resources Pharmaceutical needs among population served Low patient adherence to plan of care due to stigma and family capacity
**Inner Setting**		
*CFIR Constructs*	*Facilitators*	*Barriers*
*Structural Characteristics:* components that support functional performance, including staffing and the overall workplace environment that impacts each site	Adaptability of new care model Buy-in from hospital administration Interdisciplinary, team-based approach to care Familiarity following clinical protocols and guidelines	Variability in hospital layout and adaptation needs Staffing needs for specialized care (OT/social work) Differences in specialized training between staff Inconsistent scheduling of trained staff Existing practices, processes, and policies
*Networks and Communication:* The various formal and informal information sharing (networks and communication) across the inner setting boundaries	Buy-in from hospital administration Development of a coordinated plan of care Identified point-person (and champions) for implementation and communication efforts Communication	Frustration with existing communication practices Need for reactive care in times of unanticipated challenges Gaps in communication from hospital administration Inconsistent EMR use Variations in staff scheduling
*Culture:* The shared values and beliefs across the inner setting	Aligns with organizational values Support between implementation staff and administration Education to address hesitancy	Financial concerns Implicit bias Hesitancy among staff
*Implementation Climate:* The total capacity for change and shared receptivity of individuals regarding a new intervention (includes relative priority)	High desire to adopt new care model Desire for consistency Close-knit “team” of staff Train-the-trainer model Familiarity with training and education tools	Limited resources Hesitancy among staff Lack of consistent practices Staff capacity for change Need for champions and buy-in Lack of education
*Readiness for Implementation:* Individual-level perspectives of implementation readiness within their respective sites	Aligns with personal and organizational values Expectation of gradual buy-in among staff Motivation to learn Resource accessibility	Physical environment adaptations Training and education needs Anxiety related to patient/family reactions Hesitancy among staff and parents
**Characteristics of Individuals**		
*CFIR Constructs*	*Facilitators*	*Barriers*
*Knowledge and beliefs about the intervention:* Individual’s attitudes towards intervention, and overall familiarity with the intervention	Prior exposure to intervention and related concepts Aligns with personal and organizational values Trust in evidence-based intervention Motivation to learn Resource accessibility	Little to no familiarity with intervention Individual biases Hesitancy to interdisciplinary collaboration
*Individual stage of change:* Each participant’s phase in progressing towards use of the intervention	General optimism and excitement towards intervention Motivation to learn Engagement of early adopters	Learning curve and need for support staff Hesitancy among staff Anticipation of increased workload
*Other personal attributes:* Personal traits such as values, motivations, and capacity to implement	Alignment with personal values Improved job satisfaction Meaningful roles Understanding of programmatic goals	Hesitancy among staff
**Process**		
*CFIR Constructs*	*Facilitators*	*Barriers*
*Planning:* How early methods of behavior and tasks for implementation are developed Engaging:	Champions Shared understanding of education and program importance Technological support (i.e., order set) and ongoing monitoring	Staff hesitancy Limited leadership engagement Lack of parental involvement Medication adherence Sensitive provider-patient relationships
*Champions:* Individuals dedicated to driving an implementation	Knowledge dissemination Champion-led training Communication	Lack of identified champions Limited leadership engagement

Table 1 provides a comprehensive summary of identified barriers and facilitators to MAiN implementation as reported during hospital staff participation in brief interviews. Questions were guided by the CFIR model and inductive coding identified key barriers and facilitators

### Innovation characteristics

#### Evidence strength

(6) Participants described the strong evidence of success associated with the MAiN program as a primary facilitator for adopting the treatment approach, particularly in its ability to standardize care across the unit. Nursing staff identified MAiN’s evidence-based approach of standardizing care to increase effectiveness and reduce suffering within the neonatal population. For example, a nurse stated:

“I love it because it is a medical model; it’s very prescribed. It’s a proactive intervention instead of a reactive one.”

#### Relative advantage

The relative advantage —of the MAiN program was frequently mentioned as a facilitator by participants. This was identified through repeated discussion of MAiN’s focus on reducing the burden of caregiving on staff, providing a straightforward framework for care delivery, and allowing the hospital to provide care to a population often transferred to a higher acuity care setting. The education provided by MAiN was also discussed, mainly its focus on reducing stigma around NOWS and emphasis on training to provide quality care. Participants also emphasized MAiN’s proactive treatment approach, with one stating:

“I think the biggest benefit is [for] the higher risk babies and getting them treated before they have all that pain. It can’t be good to start life with all that pain and how it affects them psychologically.”

#### Adaptability

The high level of adaptability of MAiN was identified as a facilitator to implementation. One physician discussed the importance of MAiN’s adaptability, including the developer’s communication regarding the individual site needs, when determining how well MAiN could be integrated into current practices:

“I’m interested in learning how MAiN fits into an outlying or community setting. I’m interested in observing that – there won’t be a pharmacy in the hospital, won’t be physical therapy or occupational therapy.”

#### Complexity

While participants were eager to implement MAiN, some identified the complexity,as both a barrier and facilitator to implementation. Specific concerns included treating higher-risk populations, implementing couplet care (one nurse caring for both mother and newborn simultaneously), and the potential difficulty of ensuring all MAiN protocols were implemented by staff who felt the model was too complex.. A primary facilitator was the interdisciplinary program's uniqueness in treating high-risk patients, as it approaches this problem comprehensively and reduces complexity through patient-centered care. Traditionally infants with NOWS would be transferred to intensive care, whereas the MAiN protocol provides care to infants within the standard nursery setting. However, some participants expressed concerns that this approach could increase the volume of high-acuity patients and the healthcare provider burden.

### Outer setting

#### Needs and resources of those served by the organization

Several participants stressed that MAiN’s ability to enable the hospital to better serve the community by offering a previously unavailable care model inclusive of mothers is a primary facilitator. One nurse stated:

“Currently, moms may feel “caught,” so they hide out and don’t come in for visits, and don’t return phone calls. Make excuses. Worry about being drug screened again and worry about what happens to the child. They come in, deliver, and then disappear for four or five weeks with no visitation. Babies can’t get better this way.”

Availability of substance use education for providers as well as available treatment for infants and families were identified as significant facilitators of MAiN implementation, with one social worker explaining:

“We do not have enough counseling programs. We do have several drug assistance programs in town, but we do not have a connection to those, so if I have a mom with needs, we do not have a place to send her.”

Some participants reported a lack of knowledge specific to community-based SUD resources and commonly used stigmatizing language around substance use among providers as barriers when communicating with families of infants with NOWS. A nurse mentioned:

“In general, we struggle with access to resources. Providing a friendlier environment for families and individuals with substance use is essential. The hospital does a poor job of this.”

### Inner setting

#### Structural characteristics

Staffing and availability to manage the new approach to patient care were reported as a primary barrier, with one nursing administrator describing:

“It can be very trying on the staff when we don’t have sufficient staff that we’d like to provide for that baby. If we have a full census and a [NOWS] baby, it really wears on the nurses. You feel bad if you have a baby that you cannot help at that time.”

#### Networks and communication

When asked about the various networks and communication across the inner setting boundaries, the lack of established pathways for referral communication with outpatient providers was a primary barrier cited by participants. However, strong and consistent communication (i.e., formal announcements versus word of mouth) early in the MAiN implementation process was a facilitator in increasing participation in implementation.

#### Culture

Discussion of culture provided an informative view of each hospital’s climate. Both hospital-wide and unit-specific cultures were frequently mentioned as both a barrier and facilitator. One participant mentioned this facilitator:

“Over the years, it’s been very open. Our physicians have been open and have participated in designing the programs. We found a lot of cooperation and enthusiasm in creating the types of protocols or management programs. It’s been easier than you would imagine, it’s more akin to what you would see at a large hospital.”

Participants frequently referred to hospital mission when asked to describe their primary motivator in implementing the MAiN implementation at their hospital, as one stated:

“We want to be the number one choice of healthcare in our area, and we are making progress in that direction. We are having a hard time keeping up with our growth. All these services and this program will just be another feather in the cap showing the community that we could be their choice for healthcare.”

#### Implementation climate

The implementation climate at each hospital was viewed positively. Most participants expressed that this new model of care would be implemented quickly and without significant challenges, as a new approach was needed to treat the increasing rate of NOWS. A nurse mentioned:

“It fits completely, this is a problem is here whether we approve or not, and needs a solution, and we should be part of that solution.”

Many respondents reported that early buy-in and ensuring adequate staff education to use this approach would be critical facilitators. The relative advantage of the program was often mentioned in conjunction with the implementation climate, with many participants stating that the unique benefits of MAiN would facilitate a positive implementation process. A barrier consistently related to compatibility was that of the nursing staff’s ability to care for this higher-risk population. One nurse mentioned the following barrier:

“Overall, for me personally, as a mom and caregiver, if I’m helping that patient, it helps me because we hate seeing these babies suffer, any kind of intervention that is going to help them is going to benefit me and other coworkers.”

Relative priority—the shared perception of implementation importance [6]—in implementing MAiN was emphasized by some participants stating that the hospital had recognized the increasing numbers of these infants and the need to care for the NOWS population. A nurse mentioned this facilitator:

“I do not agree with our current system of care for these babies—we want to change how we are managing them (separating mom and baby), and I like the idea of decreasing the length of stay, and this is a more humane way of treating the baby.”

However, barriers falling under implementation climate varied between the disciplines of health care providers, with many nurses stressing that responsibility for implementation would fall primarily on physicians as the diagnosticians, which could reduce the overall buy-in. One shared:

“I would say moderate difficulty. Based on bringing all different resources together. Nurses will do well with training, but our doctors are not as proactive and may resist training. Not because they don’t want to learn but because they don’t have time.”

#### Readiness for implementation

Participants from all seven hospitals identified a primary champion to support the MAiN implementation. Participants explained that they had prepared for the initial site visit through education and organized the visits. The administration engagement at each hospital was also reported to be a key to successful implementation. A significant barrier to readiness was the availability of resources, or lack thereof, with some participants being concerned about the hospital’s ability to provide rooming and centralized infant monitoring.

### Characteristics of individuals

#### Knowledge and beliefs

There was variation in knowledge of the MAiN program, which can be either a barrier or a facilitator depending on the level of expertise. Some participants reported being unaware, while at other sites, participants who had been connected to the developer or connected through other state-wide educational programs were aware and excited about MAiN. Several participants at the sites were able to identify the core components of MAiN, indicating the program’s substantive reach across SC.

#### Individual stage of change

Predominantly, participants were excited about incorporating MAiN into their hospital setting. The primary barriers of concern were at-home weaning protocols and ensuring that respective hospitals adequately prepare parents to care for the infants following discharge. Most participants expressed optimism about implementing the program and the potential benefits. A nurse shared:

“I am hopeful because I do feel that a lot of these babies, it’s a travesty that they start out this way and have a very rough start to life. In a small community hospital, it takes a while before people get on board. You get the “this is a new guideline; it’s going to change again” mentality from people. I’m hopeful because we’ve all seen firsthand how miserable babies can be.”

#### Other personal attributes

Many of those interviewed described their motivations to participate, wherein the importance of providing compassionate care for this population was consistently reported. As one participant explained:

“I don’t believe in allowing suffering as a physician. In the care of infants, we minimize suffering because they can’t tell us. I’ve watched infants withdraw, and it’s absolutely suffering, and this program minimizes that experience.”

Some participants voiced frustration with mothers delivering NOWS babies, and one nurse stated that they hoped this program could provide support for mothers to be involved in the infants’ care:

“Personally, I am interested in learning how to be more compassionate to these patients. Even less judgmental. I hope there are tools for all of us to understand. MAiN may reduce that stigma on how to talk to these people and how to make this easier. If they benefit in no other way, reducing stigma is worth it.”

### Process

#### Champions

Clinical sites with identified champions have an increased likelihood of successful implementation [11] as champions facilitate implementation. At sites that identified at least one champion for MAiN, nurses were most frequently identified as filling that role. Some participants indicated that champions were more proactive in changing the policies for NOWS treatment and pushed for more standardized care approaches even before the possibility of MAiN implementation. They championed this by directing funding, applying to participate in the MAiN program, and increasing education and awareness training for providers at the hospital. One unique aspect of the champion role was hospital-specific – at each site, either the pediatric or nursing staff was the primary driver, not both.

#### Planning and engaging

Many hospitals reported low communication from hospital leadership about MAiN before the initial site visit, a barrier to the initial implementation. When asked about the best way to plan for implementation, one provider said:

“Start two years away from doing it. I think it is good if everyone’s role is understood. That is one of the big problems; you must use the will up front to try to pick champions. One of the main reasons I have done this is because I know [the pediatrician].”

When it came to planning and engaging external providers, which remains a major barrier to perinatal treatment for women and their infants at risk of NOWS,,, another physician stated:

“We also need to reach out to the OB and fetal medicine groups of moms that are going to deliver here and let them know about the program. Even if it’s dropping flyers in the office. I must be the bad guy because moms come in here thinking one thing and then baby has to stay for five days. Getting the conversation started earlier will be needed.”

## Discussion

We identified barriers and facilitators to the readiness of seven SC hospitals to implement an evidence-based treatment model for managing infants with NOWS. Identified facilitators included the importance of having an evidence-based care model for treating NOWS, the opportunity for mothers to be involved in caring for infants with NOWS, and program champions on-site at each facility. Relevant barriers included communication strategies of each hospital site, knowledge regarding the program itself, and the need for external to the hospital resources, such as occupational therapy, social services, and substance use support centers.

### Innovation characteristics

The importance of an evidence-based care model for treating NOWS positively influenced providers’ willingness to adopt the MAiN program, consistent with other studies examining the importance of evidence-based care adoption in clinical settings [12]. In particular, nurses were keenly interested in a more structured, consistent, and patient-centered approach to NOWS care, highlighting the clinical needs of a population rarely consulted in developing new NOWS treatment approaches [13–15]. The relative advantages of MAiN, including its packaged NOWS-centered education and focus on compassionate care, emphasizes the importance of reducing stigmatizing practices surrounding substance use in healthcare [16]. A primary barrier within the innovation characteristics domain centered around the program’s complexity. Several staff members, particularly nurses, were unsure of how complex the primary approaches outlined by MAiN would be to implement, as many were not primarily employed in higher-acuity settings like neonatal intensive care units. Moving forward, it is vital to ensure that staff are fully trained to reduce complexity-related issues or concerns during implementation.

### Outer setting

Beyond the acute care setting, participants expressed a desire for an improved hospital connection to community resources once infants and families have been discharged. A primary driver for participation in MAiN was the improved care that could be delivered to the community each hospital served. Traditional NOWS care limits parental involvement in infant care beginning at birth. Nursing staff want to change how parents can participate in early care for infants, ultimately improving bonding and increasing knowledge about the needs of infants with NOWS among families [17, 18]. Overall, there was significant variation in how their children’s healthcare providers treated mothers. Implementing the MAiN program provides support and allows mothers to understand the needs of infants with NOWS and feel more engaged in their infant’s care [19]. Each site also had unique community resources for patient referrals and limited support for families of NOWS infants following discharge. However, several providers lacked knowledge about available resources for families following discharge, indicating a need for improved communication beyond the hospital’s walls. MAiN education can enable providers with compassionate, de-stigmatizing language and communication training, which is necessary to improve early initiation and continuation of care during pregnancy and following delivery among women currently using opioids [20].

### Inner setting

Each hospital had unique factors that both facilitated and hindered the potential for implementation success. Cultural environments, including staff’s willingness to participate and the perceived difficulty of implementing a new care delivery model, heavily influenced the implementation climate and subsequent enthusiasm at each site. Many participants identified staff hesitancy and resistance to changing care protocols for this population. On the other hand, early adopters, or champions, were identified as facilitators to bypass resistance and increase buy-in across disciplines. These findings are consistent with previous research that identified cultural climates as a major factor in implementing interventions [21, 22]. Communication strategies used at each site were also a major barrier or facilitator, depending on the quality and style of the strategy employed. Hospitals where participants reported consistent communication indicated that communication style could be a critical facilitating aspect of the MAiN implementation.

### Characteristics of individuals

Because MAiN provides comprehensive, compassion-centered education in conjunction with treatment protocols for providers to streamline care, this program can potentially improve provider communication and efficacy regarding NOWS care. Our identification of individual-level provider attributes also highlights the importance of direct communication with primary stakeholders during a program's implementation phase to increase the likelihood of successful implementation [23].

### Process

Early adopters, or champions, have repeatedly demonstrated their critical role in implementation efforts across healthcare settings [24, 25]. Participants who identified strong champions at their sites expressed greater expectation that these individuals would gradually persuade others to buy in to the new model of care. This evaluation identified interdisciplinary champions who provided hands-on care, fostered a participative leadership style, and were passionate about supporting the MAiN program, consistent with other literature emphasizing the importance of clinical champions [11]. Alternatively, we found that OBGYNs were identified as having minimal engagement prior to MAiN implementation. Future methods of outreach to women’s healthcare providers could improve enrollment and subsequent involvement in NOWS care.

### Limitations

This study has several limitations. The sample was a convenience sample; predominantly nursing staff and pediatricians were interviewed. Nevertheless, our sample (*n* = 82) was large, and participants were recruited from seven separate and varied healthcare facilities statewide. Participants may have felt more comfortable discussing the adoption of a new intervention outside of their place of employment. However, due to the difficulty of conducting multiple interviews with hospital staff who work in shifts, it was deemed most feasible to conduct interviews in this manner. Still, they do provide insight and lessons learned for hospitals in other states aiming to implement evidence-based care models for NOWS. Further research is also needed to understand the longitudinal influence of cultural and individual-level characteristics on MAiN sustainability within the hospital setting.

## Conclusions

The present evaluation identified barriers and facilitators to the readiness of seven SC hospitals to implement an evidence-based treatment model for managing infants with NOWS. As with any implementation of an evidence-based program, it is crucial to consider the factors that influence implementation in a complex and dynamic hospital environment. Facilitators included MAiN’s evidence-based approach to treating NOWS, the provision of opportunities for mothers to be involved in NOWS care, and implementation champions at each facility. Barriers to implementation included the chosen communication strategies of each hospital site, knowledge of MAiN’s core components, and lack of external resources, such as occupational therapy, social services, and substance use support centers. The findings found within this analysis will inform future iterations of MAiN, with the goal of expanding beyond South Carolina hospitals. In particular, baseline evaluation helped to identify key provider groups, such as OBGYNs and local pediatric groups, who are critical providers of care for NOWS infants and their families. A focus on education and partnerships with these groups could improve NOWS outcomes and potentially reduce the burden of NOWS treatment on healthcare facilities through earlier initiation of prenatal care and improved pediatric treatment for infants with NOWS [26, 27].

Of note, this evaluation was conducted prior to the publication of the updated CFIR constructs. Following the completion of MAiN 2.0 at ten hospitals statewide, the evaluative team has plans to use the updated version of the CFIR model to improve our findings and ensure relevancy and rigor in final evaluative reports [28]. These findings have the potential to inform future implementations of care models for infants with NOWS, as well as highlighting significant barriers and facilitators to the successful implementation of evidence based NOWS programs.

## Supplementary Information

**Additional file 1.**

## Data Availability

The data that support the findings of this study are available from South Carolina Department of Health and Human Services but restrictions apply to the availability of these data, which were used under license for the current evaluation, and so are not publicly available. Data are however available from the authors upon reasonable request and with permission of the South Carolina Department of Health and Human Services.

## Abbreviations

NOWS: Neonatal Opioid Withdrawal Syndrome
MAiN: Managing Abstinence in Newborns
CFIR: Consolidated Framework for Implementation Science
