# Supplementary material for: “It’s a proactive intervention instead of a reactive one”: measuring facilitators and barriers regarding readiness to implement a treatment program for infants with neonatal opioid withdrawal syndrome
Source: BMC Health Serv Res. 2023 Jul 14;23:754. doi: 10.1186/s12913-023-09734-8 (PMC10347713; doi:10.1186/s12913-023-09734-8)
Supplement: Supplementary file 1 — Additional file 1. [file 12913_2023_9734_MOESM1_ESM.docx]

**Appendix A.**

MAiN 2.0 Implementation Evaluation
Baseline Data Collection Tool

1. Why did your hospital decide to participate in MAiN?
2. Can you tell us more about your hospital’s need for this program? (CLIMATE: TENSION FOR CHANGE)
   1. *Probes:*
      1. *need for training in NOWS care?*
      2. *high rates of NOWS at hospital? in county?* (PATIENT NEEDS/RESOURCES)
3. Based on your knowledge, what do you believe are the benefits and barriers to implementing MAiN in your hospital.
4. What are the benefits of implementing the MAiN model? (EXECUTING)
   1. For you?
   2. For hospital staff?
   3. For patients and families?
5. What are the barriers to implementing the MAiN model? (EXECUTING)
   1. For you?
   2. For hospital staff?
   3. For patients and families?
6. How do you perceive the level of difficulty in implementing the MAiN model? (COMPLEXITY)
7. How are other new interventions adopted/implemented in your hospital?
8. What is the organizational culture around adopting new interventions? (CULTURE)
9. How will the MAiN model be integrated into current processes in your hospital? (CLIMATE: COMPATIBLITY)
10. Are there key champions for the MAiN model at your hospital? (ENGAGING: CHAMPIONS)
    1. How have these individuals championed MAiN? (ENGAGING: CHAMPIONS)
11. Has MAiN received support from hospital and/or community leaders?
    1. How have these leaders been supportive of MAiN? (READINESS FOR CHANGE LEADERSHIP)
12. What do you currently know about the MAiN intervention? (KNOWLEDGE/BELIEFS)
13. How do you feel about the intervention being used in your hospital? (KNOWLEDGE/BELIEFS)
    1. *Probe: hesitant, willing, excited, not excited?*
14. Why are you interested (or not interested) in adopting the MAiN model? (KNOWLEDGE/BELIEFS)
    1. *Probes:*
       1. *How does MAiN fit with your hospital’s overall mission? Your personal values?* (CLIMATE: COMPATIBLITY)
       2. *How well does MAiN fit within your hospital’s existing organizational processes and practices?For example, ensuring babies and moms can room in and moms can stay with babies the entire stay, which nurses care for babies with NOWS.*
